# Anterior Dens Screw Fixation for Traumatic C1-2 Lateral Subluxation With 3-Part Fractures of the C2 Axis (Dens, Lateral Mass, and Hangman Fractures): A Case Report

**DOI:** 10.5435/JAAOSGlobal-D-21-00260

**Published:** 2021-12-09

**Authors:** Dong-Gune Chang, Jong-Hyun Ko, Jong-Beom Park, Gang-Ik Ju

**Affiliations:** From the Department of Orthopaedic Surgery, Sanggye Paik Hospital, College of Medicine, Inje University, Seoul, Korea (Dr. Chang); the Department of Orthopaedic surgery, Chonbuk National University Hospital, Jeonju, Korea (Dr. Ko); and the Department of Orthopaedic Surgery, College of Medicine, The Catholic University of Korea, Seoul, Korea (Dr. Park and Dr. Ju).

## Abstract

To report the first case of traumatic C1-2 lateral subluxation with three-part fractures of the C2 axis (dens, lateral mass, and hangman fractures) that was successfully treated with only anterior dens screw fixation.

**Methods:**

A 56-year-old man was admitted to the hospital complaining of neck and left arm pains caused by a pedestrian traffic accident. Radiologic examination revealed traumatic C1-2 lateral subluxation, type 3 dens fracture (Anderson and D'Alonzo classification), fracture of both C2 lateral masses, and type 1 hangman fracture (Levine and Edwards classification).

**Results:**

Preoperative closed reduction of the C1-2 lateral subluxation was successfully achieved by skull traction using a Gardner-Wells tong. The patient underwent anterior dens screw fixation for type 3 dens fracture with posterior angulation. At the 2-year follow-up visit, good reduction of traumatic C1-2 lateral subluxation and solid fusion of all three-part fractures of the C2 axis were achieved with full range of motion and stability at the C1-2 joint. In addition, notable improvement of neck and left arm pains was achieved.

**Discussion:**

Preoperative closed reduction and anterior dens screw fixation can be considered as a less invasive and motion-preserving surgery for traumatic C1-2 lateral subluxation with three-part fractures of the C2 axis.

Fractures of the C2 axis are considered to be one of the most common traumatic injuries of the cervical spine and account for between 17% and 27% of cervical spine fractures.^1^ However, multiple fractures of the C2 axis are relatively rare, and currently, no consensus for surgical management exists.^2,3^

Several poorly defined strategies have been suggested for managing multiple C2 axis fractures.^2^ For cases with multiple C2 axis fractures, posterior or combined C1-2/3 fusion is commonly done to achieve a solid fusion, despite sacrificing the C1-2 motion, especially rotation.^1-3^ Here, we report the first case of traumatic C1-2 lateral subluxation with three-part fractures of the C2 axis (dens, lateral mass, and hangman fractures) that was successfully treated with only preoperative closed reduction and anterior dens screw fixation.

## Case Presentation

**Figure 1:**
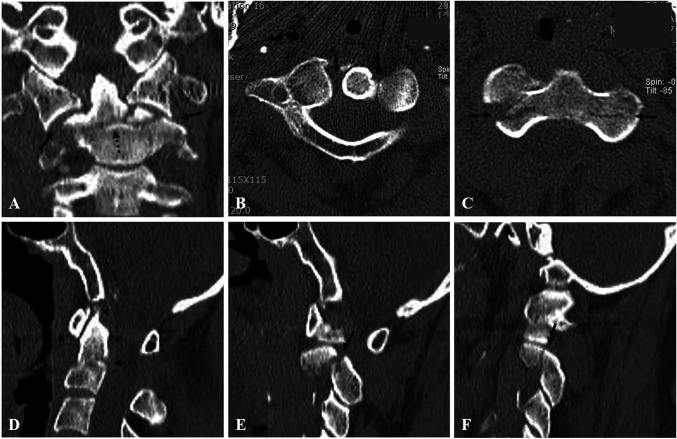
Coronal (**A**) CT (CT) scan showing a type 3 dens fracture (dotted dark arrow) and C1-2 lateral subluxation (dark arrows). Axial (**B** and **C**) CT scans showing asymmetry of C1-2 joints (dark arrows) and fractures of both lateral masses (dark arrows). Sagittal (**D**) CT scan showing a type 3 dens fracture with angulation (dotted dark arrow). Right and left parasagittal (**E** and **F**) CT scans showing fractures of both pars interarticularis (dark arrows), indicating a type 1 hangman fracture.

**Figure 2:**
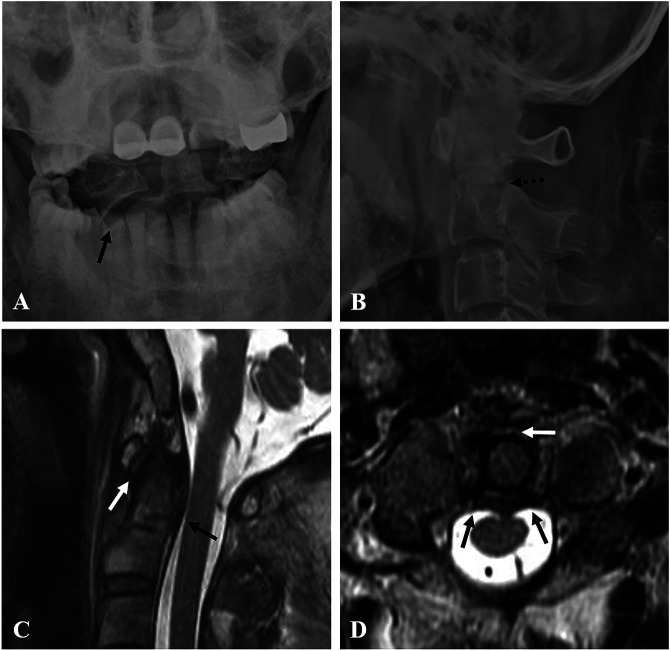
Initial open mouth view (**A**) and lateral radiograph (**B**) showing a type 3 dens fracture with angulation (dotted dark arrow) and C1-2 lateral subluxation (dark arrow). Sagittal (**C**) MRI showing a type 3 dens fracture with intact transverse atlantal ligament (dark arrow) and normal atlanto-dental interval (ADI) (white arrow). Axial (**D**) MRI showing an intact transverse atlantal ligament (dark arrows) and normal ADI (white arrow).

A 56-year-old man was admitted to the emergency department of our institution complaining of neck pain and left shoulder pain that occurred after a pedestrian traffic accident. The pain intensity for the neck and shoulder was reported at visual analog scale values of 8 and 6, respectively. Clinical examination showed tenderness and limited neck rotation. Neurologic examination showed normal motor and sensory functions of upper and lower extremities. Coronal (Figure 1, A) CT scan revealed a type 3 dens fracture (based on the Anderson and D'Alonzo classification) and C1-2 lateral subluxation to the right side. Axial (Figure 1, B and C) CT scans revealed asymmetry of the C1-2 joints and fractures of both C2 lateral masses. A sagittal (Figure 1, D) CT scan revealed a type 3 dens fracture with posterior angulation. Right and left parasagittal (Figure 1, E and F) CT scans revealed fractures of both pars interarticularis, which indicated a type 1 hangman fracture (based on the Levine and Edwards classification). Initial open mouth view (Figure 2, A) and a lateral radiograph (Figure 2, B) showed a type 3 dens fracture with posterior angulation and C1-2 lateral subluxation to the right side. Sagittal (Figure 2, C) and axial (Figure 2, D) MRI showed a type 3 dens fracture with intact transverse atlantal ligament (TAL) with a normal atlanto-dental interval.

On admission, skull traction was applied using a Gardner-Wells tong with 3 kg of weight. The weight was gradually increased to 4 kg over a period of 2 days under careful neurologic function monitoring. A serial check of a lateral radiograph and open mouth view was done to determine whether the closed reduction was successful and to prevent overdistraction of C1-2. After the closed reduction was determined to be successful, the weight was reduced to 2.5 kg for maintenance. Afterward, he underwent anterior dens screw fixation for type 3 dens fracture with posterior angulation using a 4.0-mm cannulated screw through a standard C5-6 transverse skin incision. After surgery, he wore the Philadelphia brace for 3 months.

**Figure 3:**
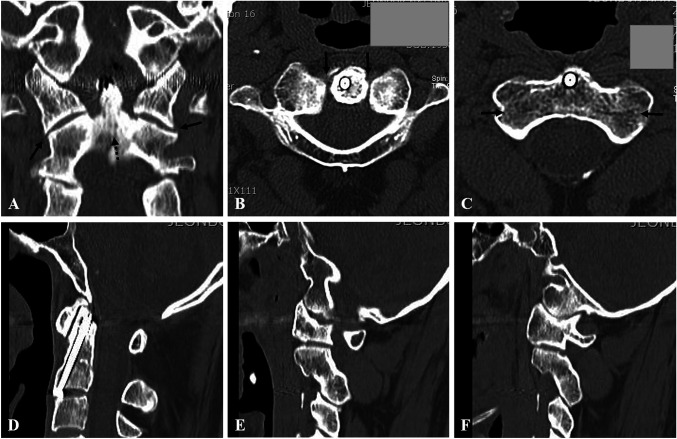
At 2 years after surgery, follow-up coronal (**A**) CT scan showing fusion of the type 3 dens fracture (dotted dark arrow) and good reduction of C1-2 lateral subluxation (dark arrows). Axial (**B** and **C**) CT scans showing symmetry of the C1-2 joints (dark arrows) and union of the fractures of both C2 lateral masses (dark arrows). Sagittal (**D**) CT scan showing fusion of a type 3 dens fracture with correction of angulation. Right and left parasagittal (**E** and **F**) CT scans showing fusion of fractures of both pars interarticularis, a type 1 hangman fracture.

**Figure 4:**
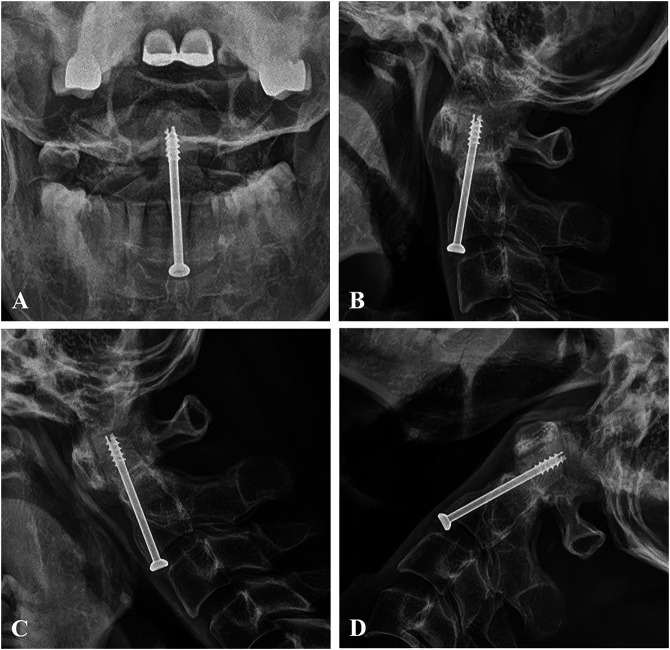
At 2 years after surgery, follow-up lateral radiograph (**A**) and open mouth view (**B**) showing fusion of the type 3 dens fracture with correction of angulation. Flexion and extension lateral radiographs (**C** and **D**) showing no instability of the C1-2 joint.

At 2 years after surgery, follow-up coronal (Figure 3, A) CT scan showed fusion of the type 3 dens fracture with good reduction of C1-2 lateral subluxation. Axial (Figure 2, B and C) CT scans showed symmetry of C1-2 joint and union of the fractures of both C2 lateral masses. Sagittal (Figure 3, D) CT scan showed fusion of the type 3 dens fracture with corrected posterior angulation. Right and left parasagittal (Figure 3, E and F) CT scans showed fusion of fractures of both pars interarticularis, which was a type 1 hangman fracture. The two-year open mouth view (Figure 4, A) and lateral radiograph (Figure 4, B) follow-up images showed fusion of type 3 dens fracture with correction of posterior angulation. Flexion and extension lateral radiographs (Figure 4, C and D) showed a full range of motion and stability at the C1-2 joint. Neck pain and left arm and shoulder pain were markedly improved to visual analog scale values of 2 and 1, respectively.

## Discussion

Multiple fractures of the C2 axis (ie, three-part fractures of the axis) have been reported as case reports, and Korres et al^4^ showed that only 1% (9 of 764 patients) had multiple fractures of the axis, which is indicative of three-part fractures (dens, lateral mass, and traumatic spondylolisthesis). To the best of our knowledge, traumatic C1-2 lateral subluxation with a three-part fracture of the C2 axis (dens, lateral mass, and hangman fractures) has not yet been reported. Thus, our case will help determine appropriate decision making for traumatic C1-2 lateral subluxation with complex three-part fractures of the C2 axis.

Although conservative treatments showed good clinical outcomes for stable three-part fractures, surgical treatment is essential for the unstable nature of three-part fractures, as shown in our case. A posterior approach was widely accepted for unstable three-part fractures because it was beneficial for stability from the fracture's reduction and fusion.^1^ However, this approach showed relatively higher mortality, and Lipson^5^ reported that posterior fixation markedly decreased the range of motion of the cervical spine by up to 50%. Compared with other unstable fractures that were managed with posterior fusion, TAL was intact, which allowed sufficient stability to be achieved and preserved the range of cervical motion from less invasive fixation, despite the unstable fracture pattern that was identified in our case. Therefore, injury of TAL could be considered as an important factor for decision making or surgical methods, particularly for case of unstable three-part fractures.

Treatment of the separate isolated fractures is a useful approach for managing complex fractures.^1,4^ In our case, the patient presented with a type 3 dens fracture with posterior angulation, fractures of both C2 lateral masses, and C1-2 lateral subluxation. Preoperative closed reduction by skull traction successfully corrected the angulation and lateral subluxation of C1-2, which improved the stability of the type 3 dens fracture. Furthermore, type 3 dens fracture has been associated with higher healing potential compared with other types (type 1 and type 2 dens fractures).^1^ Based on this information, we would expect to achieve sufficient stability through anterior screw fixation and to subsequently determine whether anterior dens screw fixation should be done to minimize complications.

Most cases of unstable three-part fractures have led to aggressive surgical procedures because traumatic spondylolisthesis of C2 causes more unstable fracture.^3^ Our case showed that a less invasive fixation method (anterior dens screw fixation) could be applied through successful preoperative closed reduction in comparison with other similar cases. Therefore, the success of preoperative closed reduction has a notable effect on determining surgical choices in unstable three-part fractures.

There are several strengths to our research study. We achieved favorable outcomes by applying less invasive and motion-preserving surgery for traumatic C1-2 lateral subluxation with dens and hangman fractures as follows: fracture site stability, early mobilization, preserved range of motion in the cervical spine, and good osteosynthesis. These results suggested that preoperative closed reduction and anterior dens screw fixation can be a useful surgical option in traumatic C1-2 lateral subluxation with complex three-part fractures of the C2 axis.

In conclusion, preoperative closed reduction and anterior dens screw fixation were associated with successful outcomes for traumatic C1-2 lateral subluxation with three-part fractures of the C2 axis (dens, lateral mass, and hangman fractures). Therefore, preoperative closed reduction and dens screw fixation can be considered as a less invasive and motion-preserving surgery.
